# Exploring the Acceptability of Text Messages to Inform and Support Shared Decision-making for Colorectal Cancer Screening: Online Panel Survey

**DOI:** 10.2196/40917

**Published:** 2023-05-05

**Authors:** Soohyun Hwang, Allison J Lazard, Meredith K Reffner Collins, Alison T Brenner, Hillary M Heiling, Allison M Deal, Seth D Crockett, Daniel S Reuland, Jennifer Elston Lafata

**Affiliations:** 1 Department of Health Policy and Management Gillings School of Global Public Health University of North Carolina Chapel Hill Chapel Hill, NC United States; 2 Hussman School of Journalism and Media University of North Carolina at Chapel Hill Chapel Hill, NC United States; 3 University of North Carolina Lineberger Comprehensive Cancer Center University of North Carolina at Chapel Hill Chapel Hill, NC United States; 4 Division of General Medicine and Clinical Epidemiology School of Medicine University of North Carolina at Chapel Hill Chapel Hill, NC United States; 5 Division of Gastroenterology and Hepatology School of Medicine University of North Carolina at Chapel Hill Chapel Hill, NC United States; 6 Division of Pharmaceutical Outcomes and Policy Eshelman School of Pharmacy University of North Carolina at Chapel Hill Chapel Hill, NC United States

**Keywords:** text messages, shared decision-making, colorectal cancer, cancer screening, mHealth, cancer, health care, marginalized groups

## Abstract

**Background:**

While online portals may be helpful to engage patients in shared decision-making at the time of cancer screening, because of known disparities in patient portal use, sole reliance on portals to support cancer screening decision-making could exacerbate well-known disparities in this health care area. Innovative approaches are needed to engage patients in health care decision-making and to support equitable shared decision-making.

**Objective:**

We assessed the acceptability of text messages to engage sociodemographically diverse individuals in colorectal cancer (CRC) screening decisions and support shared decision-making in practice.

**Methods:**

We developed a brief text message program offering educational information consisting of components of shared decision-making regarding CRC screening (eg, for whom screening is recommended, screening test options, and pros/cons of options). The program and postprogram survey were offered to members of an online panel. The outcome of interest was program acceptability measured by observed program engagement, participant-reported acceptability, and willingness to use similar programs (behavioral intent). We evaluated acceptability among historically marginalized categories of people defined by income, literacy, and race.

**Results:**

Of the 289 participants, 115 reported having a low income, 146 were Black/African American, and 102 had less than extreme confidence in their health literacy. With one exception, we found equal or greater acceptability, regardless of measure, within each of the marginalized categories of people compared to their counterparts. The exception was that participants reporting an income below US $50,000 were less likely to engage with sufficient content of the program to learn that there was a choice among different CRC screening tests (difference –10.4%, 95% CI –20.1 to –0.8). Of note, Black/African American participants reported being more likely to sign up to receive text messages from their doctor’s office compared to white participants (difference 18.7%, 95% CI 7.0-30.3).

**Conclusions:**

Study findings demonstrate general acceptance of text messages to inform and support CRC screening shared decision-making.

## Introduction

The undisputed importance of shared decision-making (SDM) to the ethical engagement of patients when they “arrive at a crossroads of medical options” has led some to call SDM the pinnacle of patient-centered care [1]. At its core, SDM is an interactive process where patients and providers reach a decision by sharing the best available evidence and patient preferences when considering care options [2]. Innovative and diverse approaches are needed to engage patients in health care decision-making and to support equitable SDM. Many health care organizations now use patient portals to provide patients with personalized health-related information. However, only 15%-30% of patients use these platforms [3], with well-documented racial and socioeconomic disparities [4-7]. Based on data from 2021, most Americans now own a cell phone (97%) [8], including smartphones (85%). Furthermore, people aged 50 years and older send and receive an average of 16 text messages a day [8]. As cell phones and smartphones become omnipresent, text messaging could effectively reach and engage diverse individuals to support informed and shared cancer screening decisions. This is particularly relevant for colorectal cancer (CRC), where multiple evidence-based screening modalities (ie, colonoscopy screening, computed tomography-colonography, sigmoidoscopy, fecal immunochemical test DNA, or stool testing) are available but remain underutilized [9-11].

A prior review highlighted the predominance of text message–based interventions among mobile health interventions to improve cancer screening and early detection [12]. Multiple studies, including two systematic reviews, evaluated the use of text message reminders alone or in combination with additional interventions such as providing behavioral information to improve adherence to recommended CRC screening (eg, [10,13-18]). Similarly, multiple studies have explored the use of text messaging to support colonoscopy attendance and adequate bowel preparation in the context of CRC screening [19-22]. At least one of each of these types of studies successfully targeted people who have been historically marginalized because of racism or language barriers [17,22]. Additionally, there are ongoing research networks at the National Cancer Institute—Accelerating Colorectal Cancer Screening and Follow-Up Through Implementation Science—that aim to improve CRC screening, follow-up, and referral among underserved groups that have low CRC screening rates using a variety of approaches, some of which may include the use of text messaging. However, to our knowledge, no prior study has explicitly explored how a text message intervention might facilitate shared and informed decision-making at the time of cancer screening. We are, however, aware of one such study among patients undergoing total joint arthroplasty, which found a positive relationship between perioperative text message communications and patient reports of SDM [23], as well as two ongoing studies that are both being conducted within other clinical contexts [24,25].

Patients increasingly desire technology options that allow them to ask questions and receive health information [26,27]. Text messages can address patient questions to overcome barriers when not in the physical presence of a health care provider [28], and ultimately could encourage cancer screening and other preventive services, perhaps even among those who historically have not engaged with patient portals.

Despite strong evidence that CRC screening reduces overall CRC-related morbidity and mortality, patients are infrequently offered a choice among available tests, notwithstanding evidence that recommending one screening modality (eg, colonoscopy alone) reduces CRC screening adherence [29,30]. Offering patients SDM for CRC screening decisions could facilitate patients’ awareness of testing options and screening adherence. 

In this study, we evaluated the acceptability of text messages embedded with SDM support for CRC screening among categories of people who have been historically marginalized as defined by low income, low literacy, and Black/African American race.

## Methods

### Setting and Study Sample

Participants were recruited from an online panel of US adults maintained by a commercial online health survey company (Lightspeed, a division of Kantar), which issues points and offers prize draws to panel members for completing surveys. To be consistent with the published United States Preventive Services Task Force guidelines for CRC screening among average-risk adults at the time of the study [9], study eligibility was limited to panel members who reported being aged 50-75 years and having no personal history of cancer. We also limited the sample to those who consented to study participation and provided a working cell phone number. For the analyses, we further limited the sample to those who (1) completed an online screener questionnaire, (2) interacted with the text message program, and (3) responded to at least one question on an online postsurvey. To ensure diversity of the study sample, we used sampling quotas to ensure that half of the study sample were (1) Black/African American or Asian/Other (eg, Asian Indian, Chinese, Filipino, Japanese) race and (2) had no history of CRC screening. Data were collected from July 2020 to August 2020.

### Procedure

The study was advertised to Lightspeed Health panel members via email. Those who were interested in participating completed an online screener to determine study eligibility. Once deemed eligible, respondents were sent an online study consent form and asked to provide a valid US cell phone number. Those who consented and provided a working cell phone number were delivered experimental decision-support message content regarding CRC screening and screening test options via text message. Participants were randomly assigned to one of three experimental conditions: General Support, Doctor’s Office Support, and Standard. Participants’ responses to program-embedded questions and branching logic determined what and how much program content was sent to them. The speed with which a person completed the text message program depended on their own responses (which guided what content was pushed to them). Although the length of time it took for participants to read and respond to a received text message also varied, the program was designed to be completed in one sitting followed by the postsurvey. However, study participants were not limited to one sitting and faced no time constraints on engagement with the text message(s). Upon program completion, participants were provided with a link to an online postintervention survey.

### Text Message Content

The text message program offered educational information on CRC screening intended to address three of the most common components of SDM [31] that have been advocated as critical to its implementation in practice: choice awareness, option awareness, and decision-making [32]. The program initially provided information on who should be screened and descriptions of available screening tests (ie, colonoscopy and stool testing) to create decision or choice awareness. The content of the program also provided information regarding the testing process and the pros and cons of each test to describe treatment options and facilitate option awareness. Finally, the program prompted the user to talk to their doctor about CRC screening and which screening test might be right for them (ie, supporting making the decision). Within each section of the program (choice awareness, option awareness, and decision-making), users were prompted to input questions they might have and asked if they would like to continue or stop receiving messages.

**Figure 1:**
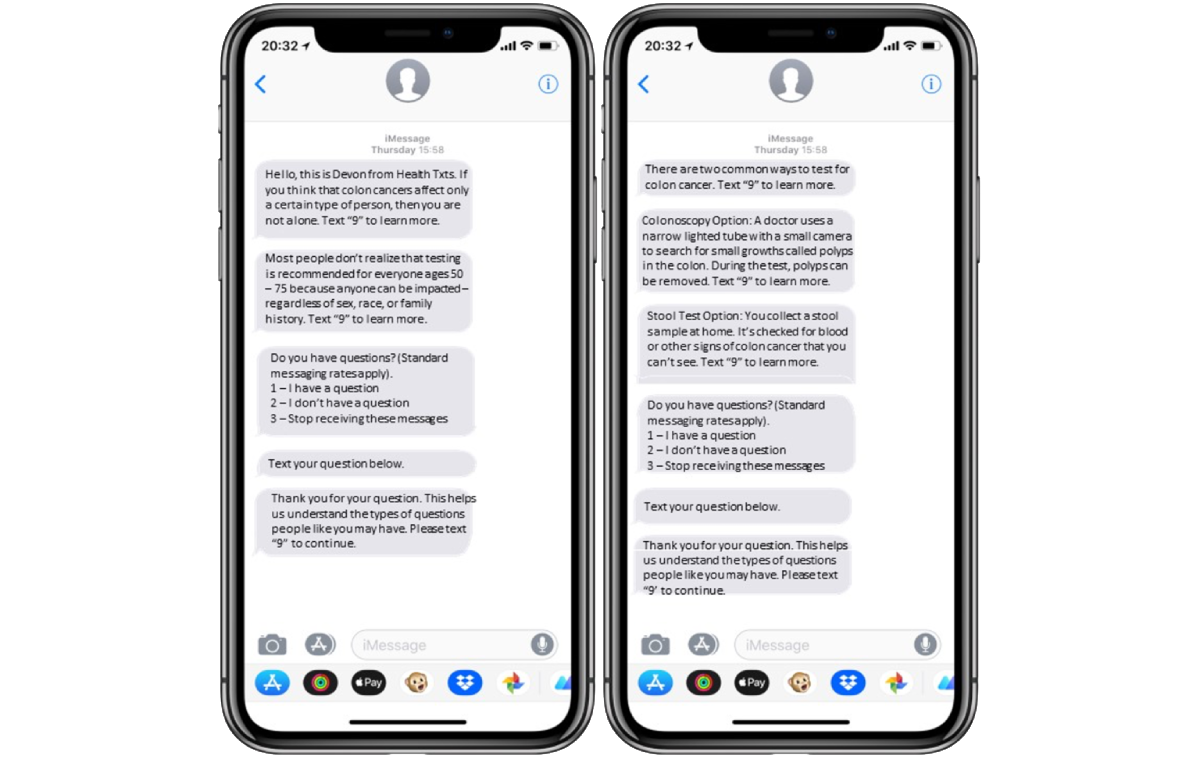
Example text messages.

Text message content was identical across experimental conditions except for the two introductory messages (see Multimedia Appendix 1). Based on the types of introductory messages, the three experimental conditions were General Support, Doctor’s Office Support, and Standard. Figure 1 shows text message examples appearing on the cell phone screen. According to prior analyses identifying no differences in any measure of acceptability by experimental condition, we considered all participants regardless of their experimental condition for the current analyses.

### Data Sources

Study data were derived from (1) the online presurvey administered prior to initiating any interaction with the text message program (ie, eligibility screener questions), (2) the online postsurvey accessed via a link provided within the final text message received, and (3) program metadata. The presurvey included questions inquiring about the participants’ sociodemographic characteristics (eg, age, race), history of cancer, and screening history. Regarding race, respondents were asked in the survey to indicate the racial categories that pertained to them among 14 different options (eg, white, Black or African American, American Indian or Alaska Native, Asian Indian, Chinese, Filipino, Japanese, Korean, Vietnamese, Other Asian, Native Hawaiian, Guamanian or Chamorro, Samoan, and Other Pacific Islander). The postsurvey was designed to assess program acceptability and other perceptions regarding the text message program. The postsurvey also included additional sociodemographic questions (eg, education, income, and health literacy). We used metadata from participants’ responses to text messages to identify where within the text message program the participant indicated they no longer wanted to receive additional messages (ie, observed program engagement).

### Conceptual Framework

The Technology Acceptance Model (TAM) suggests that a person’s use of technology can be explained by their *perceived ease of use* and *perceived usefulness*, which collectively have a direct influence on behavioral intention. Previous health care studies have used the TAM to examine acceptance of technology-based interventions such as clinical reminder system, electronic health record, and portal use among different users, including health care professionals and patients [33,34]. Due to the importance of understanding *acceptability* among those receiving health care interventions, Sekhon and colleagues [35] developed a multiconstruct theoretical framework of acceptability of health care interventions. This framework consists of constructs that conceptually overlap with the TAM (eg, ease of use and burden, perceived usefulness, and perceived effectiveness). We therefore used a similar notion of acceptability (ie, affective attitude, burden, perceived effectiveness, and self-efficacy) and incorporated the assumption that acceptability is related to behavioral intention and the actual use of the intervention (ie, program engagement) to inform and guide our research.

### Outcome Measures

#### Primary Outcomes

The primary outcome of interest was program acceptability as measured by observed program engagement, participant-reported acceptability, and participant-reported intention to use the text message program in the future.

#### Observed Program Engagement

Participants were given the option to stop receiving additional text messages at two points. The first was after being provided with information regarding the need for CRC screening and that two common screening tests (colonoscopy and stool testing) were available (ie, after the program made them aware that there was a decision to be made). The second stopping point occurred when participants were provided with an opportunity to learn more about one and/or two screening tests, but before being provided with additional information regarding the pros and cons of at least one screening method (ie, before the program provided information on available alternatives or option awareness). We used these stopping points to create binary constructs reflective of whether the participant engaged sufficiently to have (1) choice awareness and (2) information on alternatives/option awareness.

#### Participant-reported Acceptability

Participant-reported acceptability was measured with survey items mapped to a subset of constructs proposed by Sekhon and colleagues [35] (ie, affective attitude, burden, perceived effectiveness, and self-efficacy) to examine participants’ perception of the text message program. *Affective attitude* was based on the following question: “If I received this text message from my doctor’s office, I would feel: (1) supported; (2) worried.” Each had the response options of “not at all,” “a little,” “somewhat,” “quite a bit,” and “very much.” *Burden* was based on the rating of the statement “These text messages would be easy to use” responded on a 5-point Likert scale (ie, strongly disagree, somewhat disagree, neither agree nor disagree, somewhat agree, and strongly agree). *Perceived effectiveness* was based on the following statements about all the text messages they received: (1) These text messages would be useful for knowing what questions to ask my doctor; (2) These text messages would improve my ability to talk to my doctor about colon cancer testing; (3) These text messages would be useful for learning about colon cancer screening; (4) These text messages would help me make colon cancer screening decisions. The response options for these statements were the same as those used for the *burden* construct. *Self-efficacy* was based on a single statement, “Learning to interact with these text messages would be easy for me,” with the same response options on a 5-point Likert scale as mentioned above.

#### Participant-reported Intention

We measured the participant-reported intent to interact with the text messages and to sign up for this type of text message program as indicative of behavioral intention. This concept was measured using responses for the following two statements: “I would interact with these text messages if from my doctor” and “I would sign up to get messages like this from my doctor’s office.” The responses to these statements were similarly rated using a 5-point Likert scale ranging from strongly disagree to strongly agree.

### Statistical Analyses

We present descriptive statistics for study participant demographics. As mentioned above, the outcomes of interest were all rated using a 5-point Likert scale ranging from strongly disagree to strongly agree. To improve interpretability, we dichotomized these outcomes into agree (answered “agree” or “strongly agree”) and disagree (remaining scales). We used 95% CIs to describe the difference in percent agreement between races, health literacy levels, and household income levels. Multivariable modified Poisson analyses [36] were used to calculate adjusted relative risks of agreement by participant race, health literacy, and household income while adjusting for age, experimental condition, residential area, and educational attainment; these patient characteristics were selected for inclusion in final regression models based on previous literature on disparities of portal use [4-7] and bivariate associations with the outcomes. Because multivariable results did not alter the results or conclusions, we only present bivariate results.

Differences by experimental condition were evaluated using Wilcoxon rank-sum tests for continuous participant characteristics and Fisher exact tests for categorical characteristics. All analyses were performed using SAS software (version 9.4). All statistical tests were 2-tailed, with the probability of a type I error set at *P*<.05 and no adjustments for multiple comparisons.

### Ethical Considerations

The study protocol was reviewed and approved by the Institutional Review Board at the University of North Carolina, Chapel Hill (21-1417). Online informed consent was obtained from all participants before their enrollment in the study.

## Results

### Sample Characteristics

Participant characteristics (N=289) are detailed in Table 1. The mean age was approximately 60 years. Nearly 70% of participants were male, 39% were white, 51% identified as Black or African American, and 10% identified as Asian or other minority race (eg, Asian Indian, Chinese, Filipino, Japanese). Due to the small number of study participants reporting a race other than Black/African American or white, we elected to focus on Black/African American versus white comparisons when considering participant race. Most participants were confident in their health literacy, with approximately 60% expressing extreme confidence. Nearly 40% of participants reported an annual income less than US $50,000. Most participants were educated, with more than three-quarters receiving more than high school education. Slightly over one-quarter of the participants reported never having been screened for CRC.

**Table 1:** Baseline characteristics of the study sample (N=289).

Characteristics			Value
Age, mean (SD)			60.0 (6.55)
**Gender identity, n (%)**			
	Male	196 (69.0)	
	Female	87 (30.6)	
	Other	1 (0.4)	
**Race, n (%)**			
	White	113 (39.1)	
	Black/African American	146 (50.5)	
	Asian/Others	30 (10.4)	
**Residential area, n (%)**			
	Urban	80 (28.3)	
	Suburban	152 (53.7)	
	Rural	51 (18.0)	
**Health literacy (confidence), n (%)**			
	Less than extreme	102 (36.0)	
	Extreme	181 (64.0)	
**Household income (US $), n (%)**			
	<50,000	115 (40.6)	
	≥50,000	168 (59.4)	
**Educational attainment, n (%)**			
	High school or less	34 (12.0)	
	Above high school	249 (88.0)	
**Screening history, n (%)**			
	Yes	209 (72.3)	
	No	80 (27.7)	
**Type of introductory messages, n (%)**			
	General support	96 (33.2)	
	Doctor’s office support	103 (35.6)	
	Standard	90 (31.1)	

### Observed Program Engagement

Almost 84% of participants engaged with the text message program long enough to receive information on multiple ways to be screened for CRC (choice awareness), but only 39.4% engaged with the program long enough to learn about the pros and cons of at least one CRC screening modality (alternative pros/cons or option awareness). We found no significant differences in either measure of observed program engagement by participant race, health literacy, or screening history (Table 2). However, compared to participants who reported lower incomes, participants with an annual household income of US $50,000 or more were more likely to engage with the program long enough to learn there is a choice regarding CRC screening modality (choice awareness).

**Table 2:** Observed program engagement by participant race, income, health literacy, and screening history (unadjusted) (N=289).

Participant characteristic		Choice awareness	Option awareness (alternative pros/cons)
**Race**			
	Black/African American, n (%)	121 (82.9)	63 (43.2)
	White, n (%)	95 (84.1)	36 (31.9)
	Unadjusted difference, % (95% CI)	–1.2 (–11.1 to 8.7)	11.3 (–1.3 to 23.8)
**Household income (US $)**			
	<50,000, n (%)	90 (78.3)	44 (38.3)
	≥50,000, n (%)	149 (88.7)	69 (41.1)
	Unadjusted difference, % (95% CI)	–10.4 (–20.1 to –0.8)^a^	–2.81 (–15.1 to 9.5)
**Health literacy**			
	Less than extreme confidence, n (%)	91 (89.2)	43 (42.2)
	Extreme confidence, n (%)	148 (81.8)	70 (38.7)
	Unadjusted difference, % (95% CI)	7.5 (–1.6 to 16.5)	3.48 (–9.2 to 16.2)
**Screening history**			
	Yes, n (%)	177 (84.7)	78 (37.3)
	No, n (%)	64 (80.0)	36 (45.0)
	Unadjusted difference, % (95% CI)	4.7 (–6.2 to 15.6)	–7.7 (–21.3 to 5.9)

^a^ Statistically significant difference (*P*=.03).

### Participant-reported Acceptability

Perceived acceptability per postprogram survey items varied from 63.0% to 91.7%. Among the 289 participants, the majority indicated that the program was easy to use (n=261, 90.3%) and would not be a burden (n=265, 91.7%). Similarly, most participants reported that the program was useful for learning about CRC screening (n=254, 87.9%). Participants were slightly more varied in their reports that the program would be useful for them identifying questions to ask their physician (n=230, 79.6%) or deciding about CRC screening (n=228, 78.9%) and talking to their doctor about CRC screening (n=213, 73.7%). Substantially less participants felt supported by the program (n=182, 63.0%) and 28.4% (n=82) indicated that interacting with the program would make them feel worried. Those who did not indicate having a previous screening history reported that they would feel worried relatively more than those who had a previous screening history (difference 16.1%, 95% CI 3.0-29.1). However, only 28.4% (n=82) of those who indicated potentially feeling worried engaged with the text message program long enough to learn about the different types of screening modalities.

Black/African American participants reported that they would feel more supported than white participants if they were to receive these types of messages from their doctor’s office. Compared to white participants, Black/African American participants were also more likely to report that the text messages were useful for (1) improving the ability to talk to their doctors about CRC screening and (2) learning about CRC screening, but otherwise we did not find racial differences in participants reports of acceptability. We found no significant differences in patient-reported measures of acceptability by household income or health literacy (Table 3).

**Table 3:** Participant-reported text message acceptability by participant race, income, health literacy, and screening history (unadjusted) (N=289).

Participant characteristic			Feel supported	Not worried		Self- efficacy		Burden		Helpful question		Talking to the doctor		Useful learning		Help them decide	
**Race**																	
	Black/African American, n (%)	103 (71.0)			103 (70.6)		130 (89.7)		8 (5.5)		123 (84.8)		117 (80.7)		134 (93.7)		119 (82.1)
	White, n (%)	63 (55.8)			85 (75.2)		105 (92.9)		13 (11.5)		85 (75.2)		72 (63.7)		94 (83.2)		87 (77.0)
	Unadjusted difference, % (95% CI)	15.3 (2.7 to 27.8)^a^			–4.7 (–16.3 to 7.0)		–3.3 (–10.9 to 4.4)		–6.0 (–13.7 to 1.8)		9.6 (–1.1 to 20.3)		17.0 (5.2 to 28.7)^b^		10.5 (1.8 to 19.3)^c^		5.1 (–5.7 to 15.8)
**Household income (US $)**																	
	<50,0000, n (%)	68 (59.7)			80 (69.6)		102 (88.7)		8 (7.0)		90 (78.3)		86 (74.8)		100 (88.5)		94 (81.7)
	≥50,000, n (%)	110 (65.5)			124 (73.8)		158 (94.1)		14 (8.3)		136 (81.0)		122 (72.6)		151 (89.9)		130 (77.4)
	Unadjusted difference, % (95% CI)	–5.8 (–18.1 to 6.4)			–4.2 (–15.7 to 7.2)		–5.4 (–12.9 to 2.2)		–1.4 (–8.4 to 5.6)		–2.7 (–13.0 to 7.6)		2.2 (–9.0 to 13.3)		–1.4 (–9.6 to 6.8)		4.4 (–5.9 to 14.6)
**Health literacy**																	
	Less than extreme confidence, n (%)	61 (59.8)			68 (66.7)		92 (90.2)		7 (6.9)		83 (81.4)		78 (76.5)		91 (90.1)		82 (80.4)
	Extreme confidence, n (%)	117 (65.0)			136 (75.1)		168 (92.8)		15 (8.3)		143 (79.0)		130 (71.8)		160 (88.9)		142 (78.5)
	Unadjusted difference, % (95% CI)	–5.2 (–17.8 to 7.4)			–8.5 (–20.3 to 3.4)		–2.6 (–10.3 to 5.0)		–1.4 (–8.5 to 5.7)		2.4 (–8.0 to 12.8)		4.7 (–6.6 to 15.9)		1.2 (–7.0 to 9.4)		1.9 (–8.6 to 12.5)
**Screening history**																	
	Yes, n (%)	133 (63.9)			159 (76.1)		193 (92.3)		15 (7.2)		169 (80.9)		154 (73.7)		186 (89.9)		166 (79.4)
	No, n (%)	49 (61.3)			48 (60.0)		71 (89.9)		8 (10.1)		61 (77.2)		59 (74.7)		68 (86.1)		62 (78.5)
	Unadjusted difference, % (95% CI)	2.7 (–10.7 to 16.1)			16.1 (3.0 to 29.1)^d^		2.5 (–6.0 to 10.9)		–3.0 (–11.3 to 5.4)		3.7 (–7.9 to 15.2)		–1.0 (–13.2 to 11.2)		3.8 (–5.8 to 13.3)		0.9 (–10.5 to 12.4)

^a^Statistically significant difference (*P*=.01).

^b^Statistically significant difference (*P*=.004).

^c^Statistically significant difference (*P*=.01).

^d^Statistically significant difference (*P*=.02).

### Participant-reported Behavioral Intention

Among the 289 participants, the majority indicated a willingness to interact with similar programs from their doctor’s office (n=253, 87.5%), as well as a willingness to sign up for similar programs from their doctor’s office (n=210, 72.7%). Black/African American participants, compared to white participants, were more likely to indicate an intent to (1) interact with a similar text message program from their doctor’s office and (2) sign up for a similar program. We found no significant differences in participant-reported behavioral intention by household income, health literacy, or screening history (Table 4).

**Table 4:** Behavioral intention by participant race, income, health literacy, and screening history (unadjusted) (N=289).

Participant characteristic		Interact with the program	Sign up for the program
**Race**			
	Black/African American, n (%)	134 (92.4)	117 (82.4)
	White, n (%)	93 (82.3)	72 (63.7)
	Unadjusted difference, % (95% CI)	10.1 (1.1 to 19.2)^a^	18.7 (7.0 to 30.3)^b^
**Household income (US $)**			
	<50,000, n (%)	101 (87.8)	87 (75.7)
	≥50,000, n (%)	148 (88.1)	121 (72.0)
	Unadjusted difference, % (95% CI)	–0.3 (–8.3 to 7.7)	3.6 (–7.5 to 14.7)
**Health literacy**			
	Less than extreme confidence, n (%)	91 (89.2)	76 (74.5)
	Extreme confidence, n (%)	158 (87.3)	132 (72.9)
	Unadjusted difference, % (95% CI)	1.9 (–6.6 to 10.4)	1.6 (–9.8 to 13.0)
**Screening history**			
	Yes, n (%)	188 (90.0)	154 (74.4)
	No, n (%)	65 (82.3)	56 (71.8)
	Unadjusted difference, % (95% CI)	7.7 (–2.6 to 17.9)	2.6 (–9.9 to 15.1)

^a^Statistically significant difference (*P*=.02).

^b^Statistically significant difference (*P*=.002).

## Discussion

### Principal Findings

Among an online panel of socioeconomically diverse US adults aged 50-75 years maintained by a commercial online health survey company, we found high acceptability for the use of text messaging to inform and support SDM for CRC screening. In a subset of measures, Black/African American participants showed even greater acceptability and behavioral intention than their white counterparts. We did, however, find that participants reporting an income less than US $50,000 were less likely than those reporting higher income to engage long enough with the program to learn that multiple CRC screening tests are available (choice awareness). Our findings support promising opportunities that text messaging–based programs might enable health care organizations and others to reach broader populations than they could by relying solely on online patient portals, but nonetheless illustrate caution regarding the extent to which text messaging can be used to support components of SDM.

### Comparison With Prior Work

Many health systems have turned to online portals to deliver health education materials to engage and support SDM outside of office visits. Because of well-documented disparities in patient portal use [4-7], identifying additional communication channels to support these efforts is imperative. Consistent findings from the mobile technology and public health literature is that text messages for behavioral change (eg, weight loss) are most effective when perceived as relevant, personalized, and simple [37,38]. Our findings suggest additional evidence that text message–based programming may facilitate patients’ decision awareness regarding CRC screening and that such text message–based programming is generally acceptable to sociodemographically diverse populations. Over 80% of study participants engaged with enough of the text messaging program to receive information about multiple evidence-based CRC screening tests available. Even among participants who reported an income less than US $50,000, over three-quarters engaged with the program long enough to view content informing them that multiple types of CRC screening tests are available. This is important, as decision awareness is often underlooked in practice and, in the case of CRC screening, may drive down screening rates [29,30]. Importantly, almost three-quarters (72.7%) of participants voiced a willingness to sign up for similar programs should they be available from their doctor’s office.

The program was only partially successful in helping participants learn about the pros and cons of alternative CRC screening tests (option awareness). Only 39.4% of study participants engaged with the program long enough to view the pros and cons of at least one of the available CRC screening tests. The consequence of this is that while most participants reported that the program was useful, relatively less participants reported that it would help them to decide or communicate with their physicians.

Taken together, our findings add to the emerging understanding that SDM is not a single event but rather a multistep process consisting of multiple components [31]. This view may support a broader implementation of SDM through text messages. Our results clearly support the use of text messaging to inform people that there are multiple ways to screen for CRC (ie, choice awareness). For a subset of people, learning about the screening alternatives also seemed feasible using text messages, whereas for others, text messages may not have been useful for acquiring in-depth information (eg, pros and cons of each screening modality).

### Limitations

Our study has several limitations. First, the sample was limited to commercial online panel members whose perspectives may not reflect the broader CRC screening–eligible population, especially those who do not routinely engage online. We also did not require study participants to be actively engaged in a CRC screening decision at the time of study participation. Second, the survey questions were adapted from existing instruments [39-41] and mapped to the conceptual framework of acceptability presented here, but they may not capture all relevant constructs as acceptability is a multifaceted concept [42]. Third, while behavioral intention is highly correlated with observed behaviors [43], the extent to which our high participant-reported intent to engage with similar text message programs would translate into actual engagement in practice is uncertain. Finally, 28.4% of participants responded in the postsurvey about how continued interaction with the program would make them “feel worried.” This indicates a potential limitation of the text message program in that “worried” people, many of whom have not previously been screened for CRC, may self-select to not interact with such programs perhaps as a coping mechanism to avoid additional worry. In other words, if “worry” is a barrier to screening, text messages may not be the best platform to engage people in learning about new information such as cancer screening modalities.

### Conclusions and Future Implications

Findings from this study demonstrate the general acceptance of text messages to engage patients in decisions regarding CRC screening as well as to support SDM in the context of CRC screening. Among people who have been historically marginalized due to racism, low income, or low literacy, the use of text messaging rather than online patient portals may better support informed and shared decision-making by enhancing decisional awareness. As our study focused on an online panel to explore initial feasibility, additional research is needed to assess acceptability among the general population, as well as to consider different ways to improve the acceptability of text message programs, particularly among lower-income populations whose mobile phone plans may cap or charge per text message use.

## Appendix

Multimedia Appendix 1 Study procedure and introductory messages.

## Abbreviations

CRC: colorectal cancer
SDM: shared decision-making
TAM: Technology Acceptance Model

## Data Availability

The deidentified data from our study are available upon request. Requests for access to the data sets and custom code generated, used, or analyzed in this study should be directed to the corresponding author.
