# Supplementary material for: Exploring the Acceptability of Text Messages to Inform and Support Shared Decision-making for Colorectal Cancer Screening: Online Panel Survey
Source: JMIR Cancer. 2023 May 5;9:e40917. doi: 10.2196/40917 (PMC10199389; doi:10.2196/40917)
Supplement: Multimedia Appendix 1 [file cancer_v9i1e40917_app1.pptx]

## Slide 1
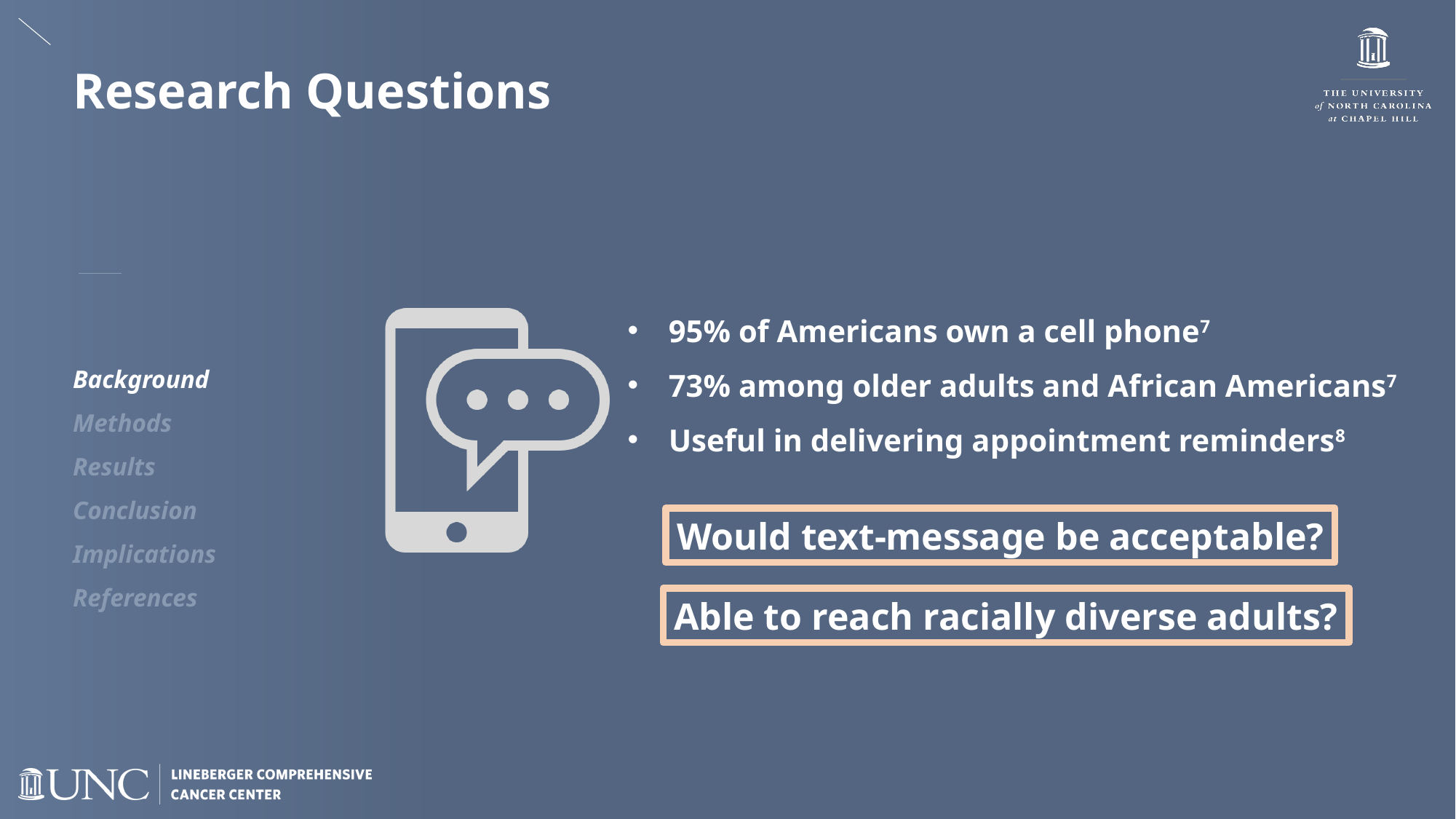

Research Questions
95% of Americans own a cell phone7
73% among older adults and African Americans7
Useful in delivering appointment reminders8
Background
Methods
Results
Conclusion
Implications
References
Would text-message be acceptable?
Able to reach racially diverse adults?

## Slide 2
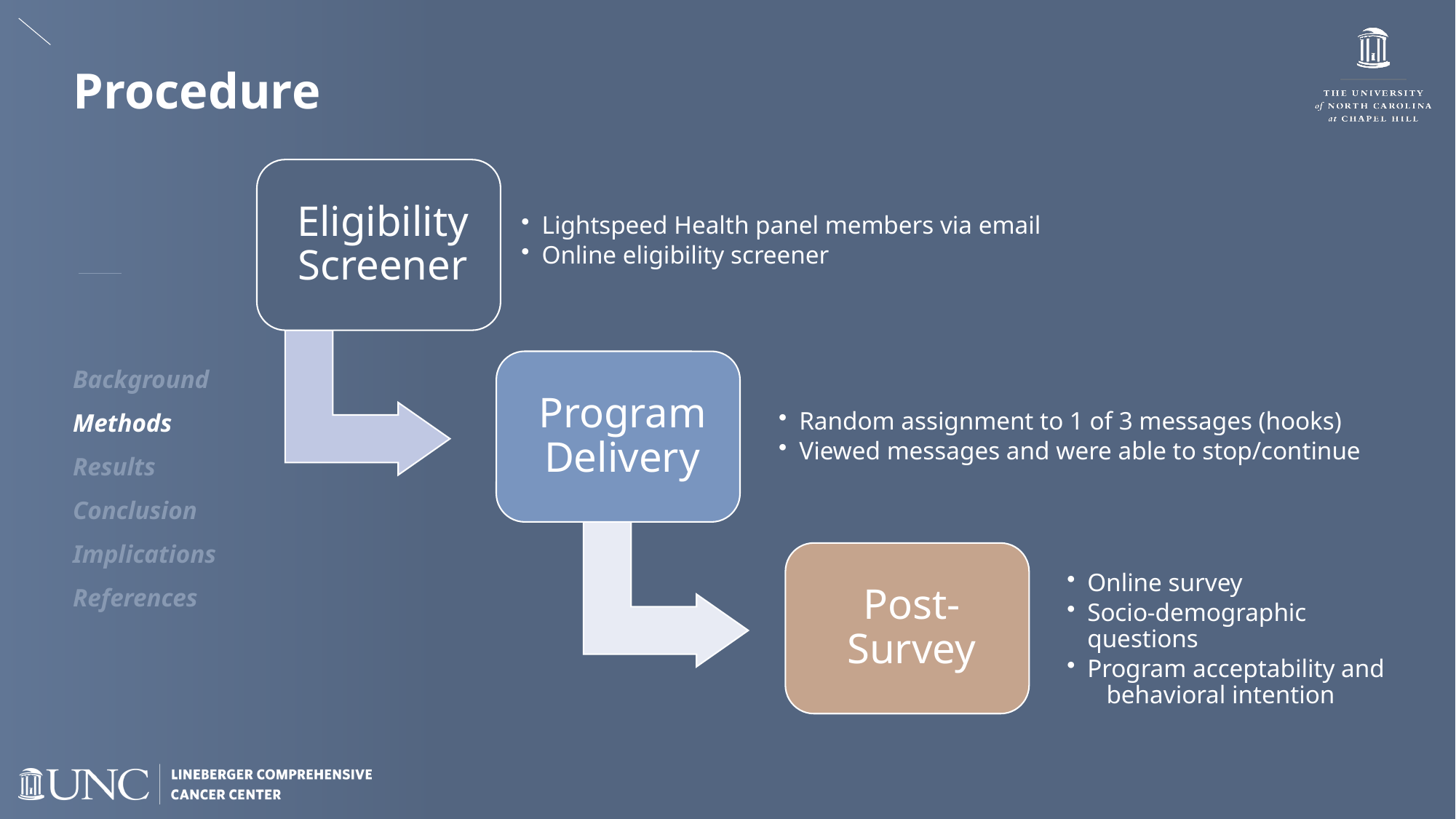

Procedure
Lightspeed Health panel members via email
Online eligibility screener
Background
Methods
Results
Conclusion
Implications
References
Random assignment to 1 of 3 messages (hooks)
Viewed messages and were able to stop/continue
Online survey
Socio-demographic questions
Program acceptability and behavioral intention

## Slide 3
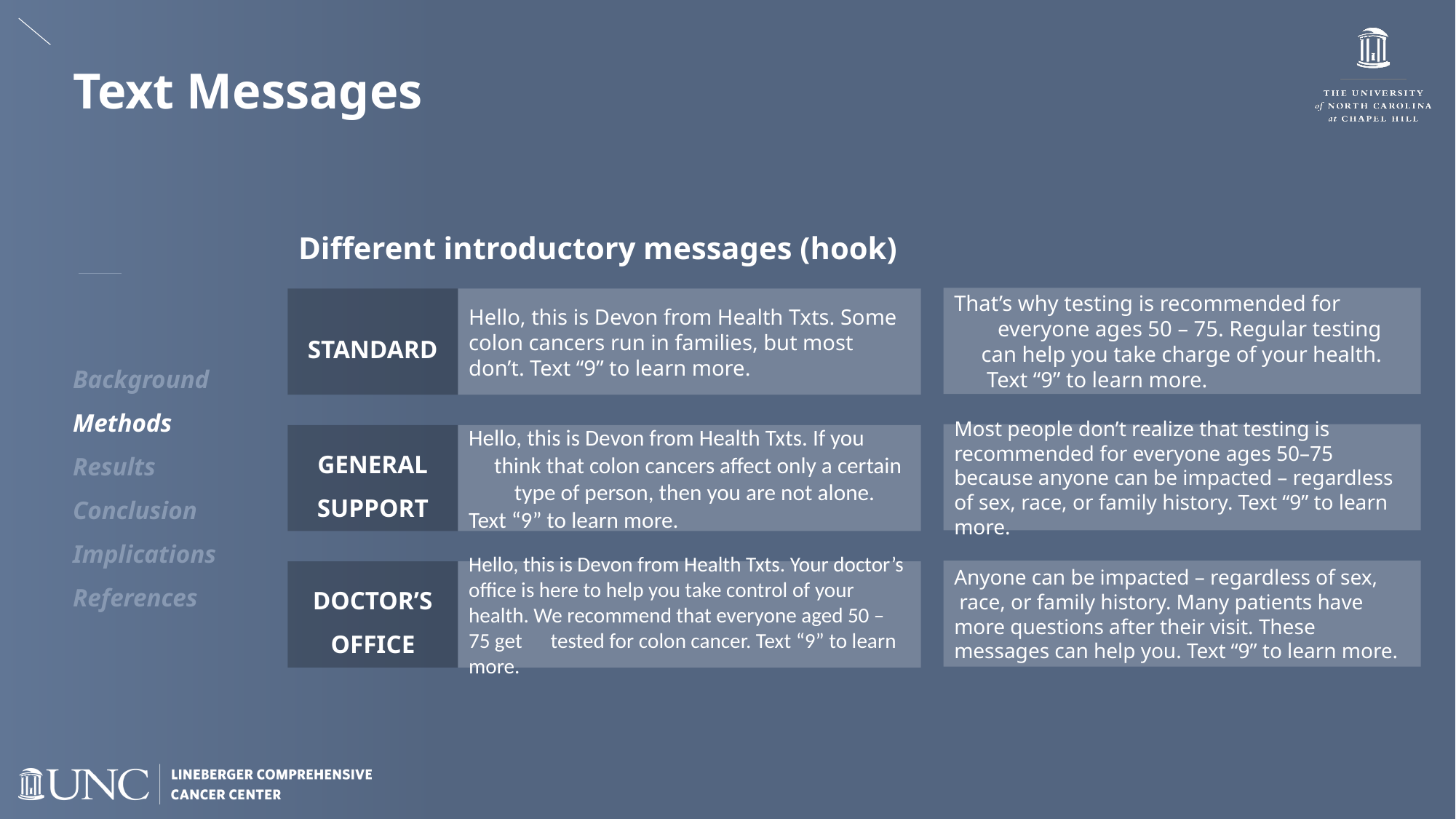

Text Messages
Different introductory messages (hook)
That’s why testing is recommended for everyone ages 50 – 75. Regular testing can help you take charge of your health. Text “9” to learn more.
STANDARD
Hello, this is Devon from Health Txts. Some colon cancers run in families, but most don’t. Text “9” to learn more.
Background
Methods
Results
Conclusion
Implications
References
Most people don’t realize that testing is recommended for everyone ages 50–75 because anyone can be impacted – regardless of sex, race, or family history. Text “9” to learn more.
GENERAL SUPPORT
Hello, this is Devon from Health Txts. If you think that colon cancers affect only a certain type of person, then you are not alone. Text “9” to learn more.
Anyone can be impacted – regardless of sex, race, or family history. Many patients have more questions after their visit. These messages can help you. Text “9” to learn more.
DOCTOR’S OFFICE
Hello, this is Devon from Health Txts. Your doctor’s office is here to help you take control of your health. We recommend that everyone aged 50 – 75 get tested for colon cancer. Text “9” to learn more.
